# Common Nodes of Virus–Host Interaction Revealed Through an Integrated Network Analysis

**DOI:** 10.3389/fimmu.2019.02186

**Published:** 2019-10-04

**Authors:** Korbinian Bösl, Aleksandr Ianevski, Thoa T. Than, Petter I. Andersen, Suvi Kuivanen, Mona Teppor, Eva Zusinaite, Uga Dumpis, Astra Vitkauskiene, Rebecca J. Cox, Hannimari Kallio-Kokko, Anders Bergqvist, Tanel Tenson, Andres Merits, Valentyn Oksenych, Magnar Bjørås, Marit W. Anthonsen, David Shum, Mari Kaarbø, Olli Vapalahti, Marc P. Windisch, Giulio Superti-Furga, Berend Snijder, Denis Kainov, Richard K. Kandasamy

**Affiliations:** ^1^Centre of Molecular Inflammation Research, Norwegian University of Science and Technology, Trondheim, Norway; ^2^Department of Clinical and Molecular Medicine, Norwegian University of Science and Technology, Trondheim, Norway; ^3^Institut Pasteur Korea, Seongnam, South Korea; ^4^Department of Virology, University of Helsinki, Helsinki, Finland; ^5^Institute of Technology, University of Tartu, Tartu, Estonia; ^6^Pauls Stradins Clinical University Hospital, Riga, Latvia; ^7^Department of Laboratory Medicine, Lithuanian University of Health Science, Kaunas, Lithuania; ^8^Department of Clinical Science, Influenza Centre, University of Bergen, Bergen, Norway; ^9^Department of Virology and Immunology, University of Helsinki, Helsinki University Hospital, Helsinki, Finland; ^10^Department of Medical Sciences, Uppsala University, Uppsala, Sweden; ^11^Department of Microbiology, Oslo University Hospital, Oslo, Norway; ^12^Department of Veterinary Biosciences, University of Helsinki, Helsinki, Finland; ^13^CeMM Research Center for Molecular Medicine of the Austrian Academy of Sciences, Vienna, Austria; ^14^Center for Physiology and Pharmacology, Medical University of Vienna, Vienna, Austria; ^15^Department of Biology, Institute of Molecular Systems Biology, ETH Zürich, Zurich, Switzerland; ^16^Centre for Molecular Medicine Norway (NCMM), Nordic EMBL Partnership, University of Oslo and Oslo University Hospital, Oslo, Norway; ^17^Program in Innate Immunity, Division of Infectious Diseases and Immunology, Department of Medicine, University of Massachusetts Medical School, Worcester, MA, United States

**Keywords:** virus–host interaction, protein–protein interaction, gene–drug interaction, innate immunity, viral evasion, network analysis, molecular innate immunity

## Abstract

Viruses are one of the major causes of acute and chronic infectious diseases and thus a major contributor to the global burden of disease. Several studies have shown how viruses have evolved to hijack basic cellular pathways and evade innate immune response by modulating key host factors and signaling pathways. A collective view of these multiple studies could advance our understanding of virus-host interactions and provide new therapeutic perspectives for the treatment of viral diseases. Here, we performed an integrative meta-analysis to elucidate the 17 different host-virus interactomes. Network and bioinformatics analyses showed how viruses with small genomes efficiently achieve the maximal effect by targeting multifunctional and highly connected host proteins with a high occurrence of disordered regions. We also identified the core cellular process subnetworks that are targeted by all the viruses. Integration with functional RNA interference (RNAi) datasets showed that a large proportion of the targets are required for viral replication. Furthermore, we performed an interactome-informed drug re-purposing screen and identified novel activities for broad-spectrum antiviral agents against hepatitis C virus and human metapneumovirus. Altogether, these orthogonal datasets could serve as a platform for hypothesis generation and follow-up studies to broaden our understanding of the viral evasion landscape.

## 1. Introduction

Viruses continue to be a major contributor to the global burden of disease through acute and chronic infections that cause substantial economic impact in addition to increased mortality and morbidity (1). Despite the tremendous improvement in the understanding of the antiviral immune response and the availability of therapeutics, existing and emerging viral diseases are an ever-growing problem, particularly in developing countries. Development of antiviral resistance of hepatitis C virus (HCV), influenza A virus (IAV), herpes simplex virus (HSV), human cytomegalovirus (HCMV), human immunodeficiency virus (HIV), and other viruses is a major concern (2–4). One of the main reasons for increasing resistances is the accumulation of mutations in the viral genome caused by multiple factors including the polymerase infidelity (5, 6). Therefore, the World Health Organization (WHO) and the United Nations have urged for better control of viral diseases.

This has led to turning the focus on the host for therapeutic intervention. Targeting the host factors has been proven to be useful for restricting viral infections (7, 8). The small molecule CCR5 inhibitor Maraviroc and the anti-CD4 monoclonal antibody Ibalizumab are examples of successful use of host-directed therapies for combating HIV in clinic (9–11).

Viruses have evolved to evade the host antiviral response at various stages starting from viral sensing to antiviral pro-inflammatory responses (12–14). Multiple studies attempted to understand global principles of the viral evasion employed by various viruses, including dengue virus (DENV), Ebola virus (EBOV), IAV, and HIV (15–20). Global systems-level approaches including functional RNAi screens, interactome mapping technologies such as affinity-purification mass spectrometry (AP-MS), quantitative proteomics, and CRISPR/Cas9-based screens have provided unparalleled details and insights into the dynamics of host proteome in immune cells (21–24), host-virus interactome (15–17, 25, 26), and also identified important host dependency factors of various viruses (25, 27, 28). Meta-analyses of such high-dimensional datasets have been crucial for identifying novel host factors as drug targets such as UBR4 in IAV infection (29). Moreover, some of these factors represent drug targets for multiple viruses (30).

We hypothesized that combining a meta-analysis of host-virus protein-protein interactions of multiple viruses and functional RNAi screens would provide novel insights for developing broad-spectrum antiviral strategies. For this, we assembled a host-virus protein-protein interactome of 5,781 host-virus interactions (hereafter referred to as “hvPPI”) covering 183 viral proteins from 17 different viruses and 2,381 host proteins. We performed extensive bioinformatics and network analysis and integrated this dataset with genome–wide or druggable–genome RNAi screen data from published studies. This resulted in the assembly of critical nodes of viral evasion and identification of core cellular processes and druggable nodes that were verified by a drug re-purposing screen using broad-spectrum antivirals.

## 2. Materials and Methods

### 2.1. Construction of hvPPI Data, Network Analysis, and Data Visualization

Host-virus protein–protein interactions were downloaded from published studies (15–17, 25, 31–34) which included a total of 183 viral proteins, 2,381 host proteins, and 5,781 host-virus interactions. Protein identifiers were mapped to UniProt IDs. Human protein-protein interaction data was imported from BioGRID database [version 3.4.139, (35)] covering 215244 interactions. The network analysis was performed using in-house programs developed in R statistical environment (version 3.4.3, www.r-project.org) with the use of the packages SparseM (version 1.77), RBGL (version 1.52.0), and graph (version 1.54.0). Network visualization was performed in Cytoscape [version 3.6.1, (36)]. Network clusters/sub-networks were extracted using the Cytoscape plugin MCODE [version 1.5.1, (37)]. Data visualization was performed in R statistical environment and Cytoscape.

### 2.2. Gene-Set Enrichment, Protein Disorder, and Sub-cellular Localization Analysis

We performed gene-set enrichment analysis using DAVID Bioinformatics Resources [version 6.8, (38)]. For all enrichment analysis, a *p*-value cutoff of ≤0.01 was used as significant. Protein disorder analysis was performed using IUPred2A software. We used the offline version with protein sequences downloaded from UniProt. Statistical analysis of disordered region distribution was performed by Kolmogorov-Smirnov test in R statistical environment. Annotation of human proteins was mapped from UniProt ID to ENSEMBL using EnsDb.Hsapiens.v86. The index of subcellular localization of interaction partners of single viral proteins was calculated for all viral proteins with ≥ five host targets. Localization of host targets was mapped using COMPARTMENTS (39), filtered for a minimum evidence score of 3 in the knowledge channel, excluding non-experimental based localization predictions. Evidence for all protein was subsequently divided by the absolute number of host-targets per viral protein. Multiple sequence alignment was performed using Clustal X [version 2.0, (40)].

### 2.3. Integration of RNAi Screens and Drug-Gene Interaction Data

Genome-wide RNAi screen data for HCV (41) and HPV18 (42), through GenomeRNAi database [(43)– GR00197], as well as druggable RNAi screen data for HPV16 (44), VACV (45), and SV40 (46) were integrated in the existing network as Z-Scores. Drug-gene interaction data was downloaded from DGIdb and drugbank. The identifiers were mapped to UniProt IDs and then compared with hvPPI.

### 2.4. Drug Re-purposing Screen

For the HMPV NL/1/00-GFP screen, approximately 4 × 10^4^ human retinal pigment epithelial (RPE) cells were seeded per well in 96-well plates. Human non-malignant RPE cell line represents excellent model system for studying replication of many viruses including respiratory (30, 47, 48). The cells were grown for 24 h in DMEM-F12 medium supplemented with 10% FBS, 0.35% NaHCO_3_, and 100 μg/ml streptomicine and 100 IU/ml penicillin. The medium was replaced with virus growth medium (VGM) containing 0.2% bovine serum albumin (BSA), 2 mM L-glutamine, 0.35% NaHCO_3_, and 1 μg/ml L-1-tosylamido-2-phenylethyl chloromethyl ketone-trypsin in DMEM-F12. HCV screen-associated cell culture conditions are described in Kim et al. (49). The compounds were added to the cells in 3-fold dilutions at seven different concentrations starting from 50 μM. No compounds were added to the control wells. The cells were mock- or virus-infected at a multiplicity of infection (MOI) of one. After 48 h of infection, the medium was removed from the cells. To monitor cell viability, CellTiter-Glo reagent was added (30 μl per well). This assay quantifies ATP, an indicator of metabolically active living cells. The luminescence was measured with a plate reader. To determine compound efficacy, HMPV NL/1/00-mediated GFP expression was measured. The half-maximal cytotoxic concentration (*CC*_50_) and the half-maximal effective concentration (*EC*_50_) for each compound were calculated after non-linear regression analysis with a variable slope using GraphPad Prism software version 7.0a. The relative effectiveness of the drug was quantified as the selectivity index ($SI\;=\;\frac{CC_{50}}{EC_{50}}$).

Cytotoxicity and antiviral activity of the compounds against GFP-expressing HCV in Huh-7.5 cells was determined as previously described (49).

## 3. Results

### 3.1. Assembly of Host-Virus Protein-Protein Interactions

**Figure 1:**
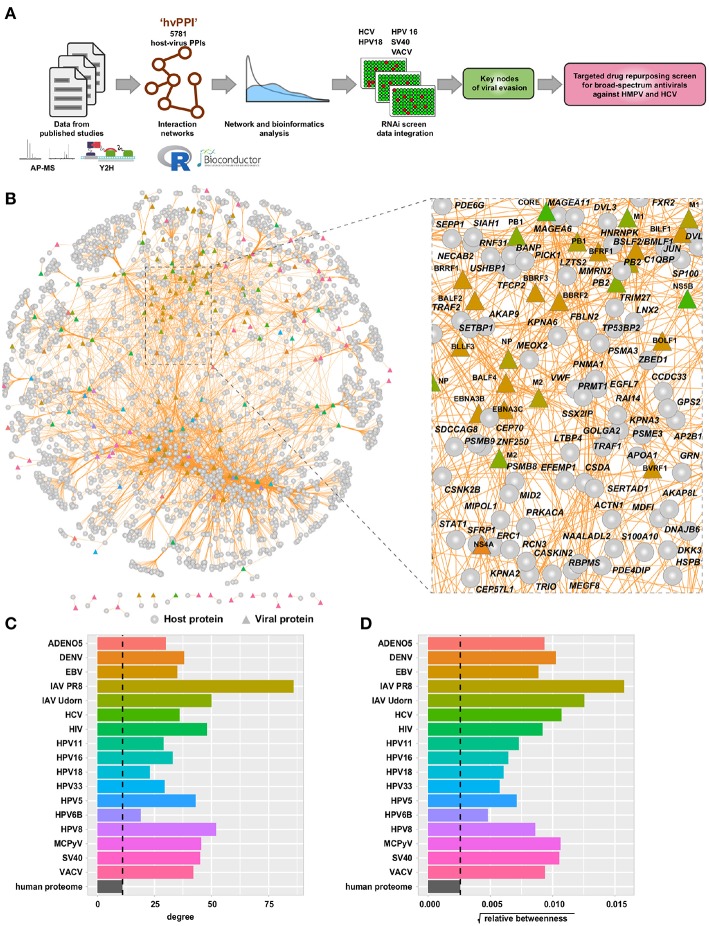
Meta-analysis of host-virus interactions involving 17 different viruses. **(A)** Schematic view of the analysis workflow. **(B)** Network view of the “hvPPI” containing host-virus interactions from 17 different viruses. The edges are colored in orange. Node shapes are in circles and triangles for host and viral protein, respectively. A zoomed-in snippet shows the names of selected host and viral proteins. **(C,D)** Barplot showing the median degree and betweenness centrality of targets of each virus as compared to the human proteome.

To provide new and critical insights into viral evasion mechanisms we performed a comprehensive meta-analysis of the host-virus interaction landscape. We assembled the host-virus protein-protein interaction data (“hvPPI”) from published studies (Figure 1A) (15–17, 25, 31–34). This dataset covered 17 different viruses including adeno-associated virus 5 (AAV5), dengue virus (DENV), Epstein-Barr virus (EBV), influenza A virus PR8 (IAV-PR8), influenza virus Udorn (IAV-Udorn), hepatitis C virus (HCV), human immunodeficiency virus 1 (HIV-1), human papilloma virus 5 (HPV5), human papilloma virus 6B (HPV6B), human papilloma virus 8 (HPV8), human papilloma virus 11 (HPV11), human papilloma virus 16 (HPV16), human papilloma virus 18 (HPV18), human papilloma virus 33 (HPV33), Merkel cell polyomavirus (MCPyV), Simian virus 40 (SV40), and Vaccinia virus (VACV). This dataset comprised of protein-protein interactions from two different types of experimental methods—affinity purification mass spectrometry (AP-MS) and yeast two-hybrid screens (Y2H). Altogether, this combined dataset includes 183 viral proteins, 2,381 host proteins, and 5,781 protein-protein interactions (Figure 1B and Figure S1). Many interactome networks including yeast and human are scale-free networks, where a large portion of the nodes (e.g., a protein in the network) have few interactions and only a few nodes have large number of interactions. The latter are often referred to as “hubs” which are crucial in keeping the network intact (34). We performed network topology analysis to infer the properties of the host proteins targeted by the viral proteins in the context of the human protein interactome. We considered two important parameters—relative betweenness centrality (which reflects the amount of information that passes through this protein in the human interactome) and degree (number of binding partners in the human interactome) of the host proteins targeted by each virus. The targets of all the viruses showed higher betweenness centrality and degree as compared to an average protein in the human interactome (Figures 1C,D). This shows that viruses, by targeting “hubs” and proteins that serves as key communication nodes, have evolved the best way to disrupt the scale-free human interactome. This topological property thereby shows how viruses having small genomes achieve the maximal effect in rewiring the human interactome to benefit viral survival and replication. Our analysis is in agreement with several previous studies, which have highlighted this property (15, 16, 31, 50, 51). We propose that this could be a general principle for all viruses.

### 3.2. Host Factors With Higher Disordered Regions Are Enriched in hvPPI Networks

**Figure 2:**
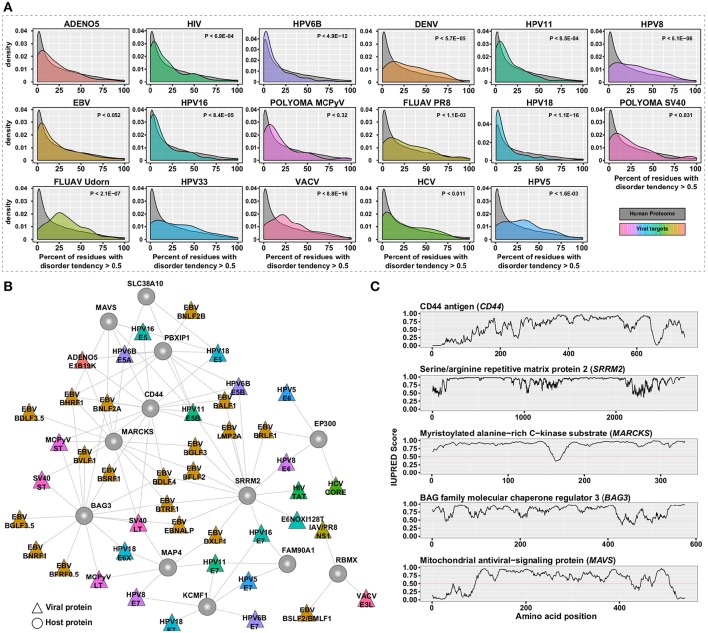
Protein disorder analysis. **(A)** Density plot of the distribution of host proteins for each virus with percent of residues with disorder tendency greater than 0.5 as predicted by the IUPred software as compared to the human proteome. **(B)** Sub-network of hvPPI with the highly targeted and highly-disordered proteins. **(C)** Line plots showing the IUPred Score (a measure of the disordered region) for the five selected host proteins from the sub-network. A IUPred score of >0.5 is considered disordered.

Proteins typically fold into stable three-dimensional structures that mediate specific functions. In addition, there are sub-structures in proteins termed “intrinsically disordered regions (IDRs)” which lack stable structures under normal physiological conditions. IDRs are required for multiple cellular functions even though they lack these defined structures (52). Many studies have highlighted the presence of such IDRs in viral proteins (53–55), such as E6 from human papilloma virus, that are crucial for hijacking the cellular machinery. We analyzed the host proteins from the hvPPI for presence of IDRs using the prediction software IUPred (56). It is estimated that the human proteome more than 100,000 short linear binding motifs in IDRs (57, 58). Proteins with IDRs are often signaling hubs and might form dynamic complexes with other proteins through specific motif based interactions (58). We found a statistically significant enrichment (*p*-value < 6.246 × 10^−06^) of IDRs in the host proteins targeted by viruses (Figure 2A and Figure S2). We then identified the subnetwork in the hvPPI which contained the top host targets with high disorderness score (Figure 2B). The top five proteins with large IDRs include CD44 antigen (CD44), Serine/arginine repetitive matrix protein 2 (SRRM2), Myristoylated alanine-rich C-kinase substrate (MARCKS), BAG family molecular chaperone regulator 3 (BAG3), and Mitochondrial antiviral-signaling protein (MAVS; Figure 2C). CD44 is a marker of exhausted CD8+ T cells (59) and replication of HCV in T cells was shown to decrease cell proliferation by inhibiting CD44 expression and signaling (60). SRRM2 is a serine/arginine-rich protein involved in RNA splicing (61). SRRM2 is differentially phosphorylated in HIV–1 infected cells and absence of SRRM2 lead to increased HIV–1 gene expression, since it regulates the splicing of HIV–1 (62). In the hvPPI, SRRM2 is targeted by multiple viral proteins including the Tat protein from HIV-1. Tat protein has an important role in the stimulation of the transcription of the long terminal repeat (LTR) (63). In addition, NS1 protein from influenza B virus has also been reported to interact with SRRM2 (64). Proteins of the MARCKS family are involved in a range of cellular processes including cell adhesion and migration (65). MARCKS is a negative regulator of lipopolysaccharide (LPS)-induced Toll-like receptor 4 (TLR4) signaling in mouse macrophages (66). MAVS is an adaptor protein in the RIG-I signaling pathway involved in the sensing of RNA. Ablasser et al. (67) reported that double- stranded DNA serves as a template for RNA polymerase II and is transcribed into a 5′ triphosphate containing double-stranded RNA, which activates the RIG-I signaling pathway. In the hvPPI, MAVS is targeted by several proteins from dsDNA viruses such as EBV and HPV. Altogether, our analysis shows that the IDR-high part of the human proteome is an essential part of the viral evasion strategy and some of the selected targets highlighted here could show novel insights into the viral evasion mechanisms. However, the very flexible protein structure of disordered proteins also makes them also difficult to target with drugs.

### 3.3. Viral Proteins Target Core Signaling Pathways and Process Networks

**Figure 3:**
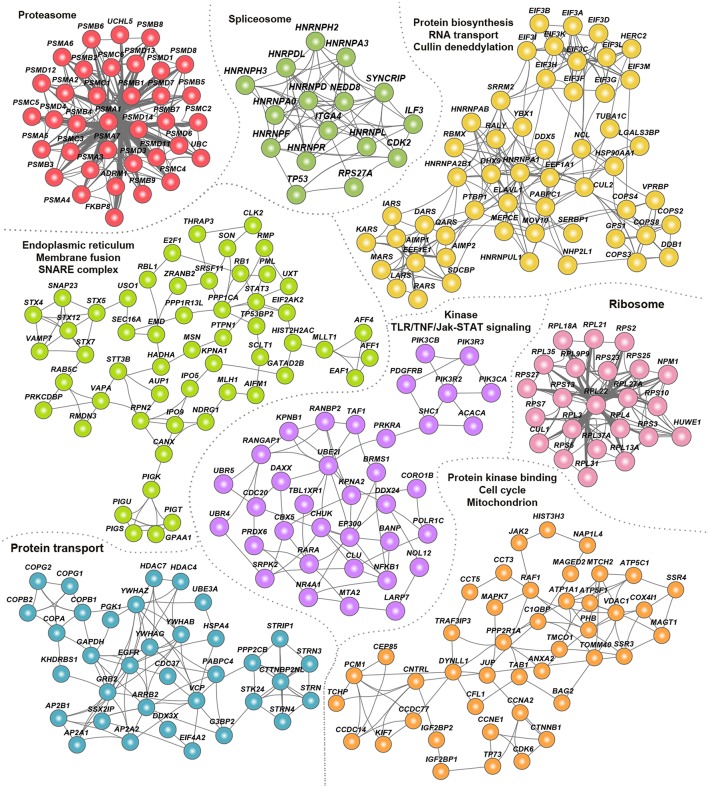
Clusters of hvPPI involved in core cellular processes. Network view of the “clusters” or highly-connected sub-networks and their associated cellular processes. Each cluster is marked in a unique color.

To assess the signaling pathways and cellular processes within the hvPPI, we identified highly connected subnetworks within hvPPI network. We constructed a host–host interaction network based on the host targets in the hvPPI and identified a number of highly connected subnetworks/clusters (Figure 3). We then performed a gene-set enrichment analysis of significantly enriched biological processes. We found one or more enriched processes for each of this subnetwork including core cellular processes such as proteasome, spliceosome, protein translation, protein/RNA transport, and cell cycle. Next we listed the viruses that target one or more of these processes, and found that almost all the core pathways and processes are targeted by all the 17 viruses that are part of the hvPPI (Figure S3). This analysis highlights the core components of the cellular process subnetworks which are targeted as part of the viral evasion strategies and thus could be broad-spectrum antiviral hot-spots from a therapeutic point of view.

### 3.4. Enrichment Analysis Reveals Commonality and Specificity in Sub-cellular Localization of the Host Factors

**Figure 4:**
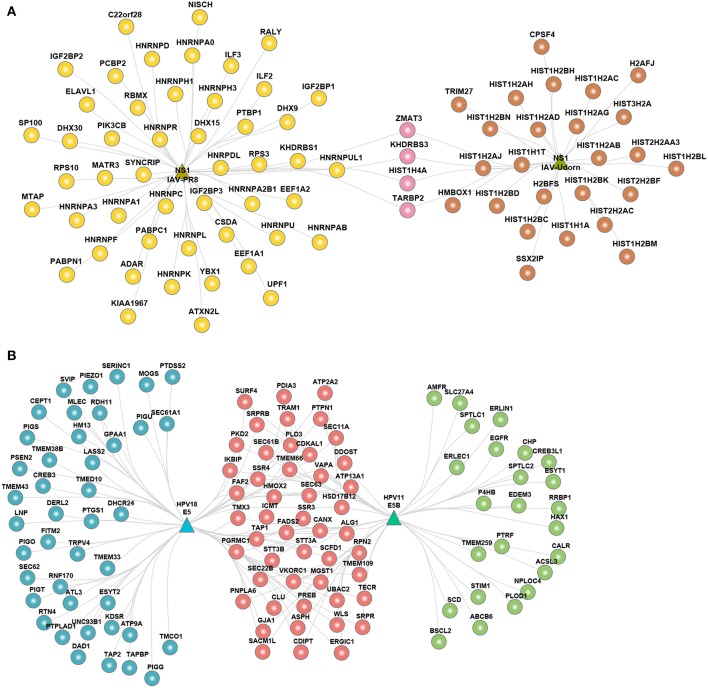
Sub-cellular localization of the host proteins. **(A)** Network view of nuclear interactome of NS1 protein from IAV strains PR8 and Udorn. **(B)** Network view of ER interactome of E5 protein from HPV18 and HPV11.

Given that the viral proteins were interacting with a large number of host proteins, we analyzed the sub-cellular location of the host proteins. We performed gene-set enrichment analysis of sub-cellular localization information provided by UniProt database. We binned the localization into 11 compartments and estimated the percent of host proteins in a given compartment as compared to the total number of host proteins targeted by a given virus. We found that the viral targets were distributed across multiple subcellular compartments with cytoplasm being the most common (Figure S4A). The hvPPI includes two different strains of IAV– PR8 (H1N1) and Udorn (H3N2). The subcellular localization analysis showed that both strains were enriched for nuclear proteins. Non-structural protein 1 (NS1) from both the strains had the highest number of nuclear targets but their targets were very different (Figure 4A). NS1 of Udorn was enriched for a large number of histones as compared to NS1 of PR8 that had large number of heterogeneous nuclear ribonucleoproteins (hnRNPs), such as HNRNPU−a known restriction factor for many viruses. This corroborates with the observation that NS1 protein has short linear histone mimicry motifs that can suppress the host antiviral response (68). In our analysis, we found that it is NS1 of Udorn that has a histone mimicry motif “ARSK” (Figure S4B). Similarly, HPV11 and HPV18 E5 proteins interact more often with host proteins located in the endoplasmic reticulum (ER). We found both common and specific subsets of ER proteins targeted by the E5 protein (Figure 4B). HPV18 E5 protein ER targets were enriched for phospholipid biosynthesis as well as GPI anchor related proteins, such as phosphatidylinositol glycan anchor biosynthesis class S/T/U (PIGS, PIGT, and PIGU), glycosylphosphatidylinositol anchor attachment 1 (GPAA1) and phosphatidylserine synthase 2 (PTDSS2). HPV11 E5 protein ER targets were enriched for ER-associated ubiquitin-dependent protein catabolism involving host proteins such as ER degradation enhancing alpha-mannosidase-like protein 3 (EDEM3) and ER lipid raft associated 1 (ERLIN1). ER targets common to HPV18 and HPV11 E5 protein were enriched for unfolded protein response, N-linked glycosylation and protein folding involving host proteins such as SRP receptor alpha/beta subunit (SRPRA/SRPRB) and catalytic subunits of the oligosaccharyltransferase complex (STT3A and STT3B). Two independent CRISPR/Cas9 screening studies identified multiple ER associated components including STT3A and STT3B as host factors for DENV, Zika virus (ZIKV) and Japanese encephalitis virus (JEV) (27, 28). The non-canonical function of STT3A and STT3B is required for DENV replication and that NS1 protein of DENV interacts with these proteins (28). Our orthogonal approach can lead to the identification of critical host factors, and similar functions of ER components, such as STT3A and STT3B, are used by HPV11 and HPV18 as well. Thus, targeting the non-canonical function of STT3A and STT3B could be a broad antiviral strategy. Overall, the enrichment analysis clearly shows that there is commonality and specificity in the subcellular targets of the viral proteins and that detailed interrogation of these targets can give vital clues into the viral evasion mechanisms.

### 3.5. Integrative Analysis of Host-Virus Interactome and RNAi Data Reveals COPI System As Commonly Targeted Proviral Process

**Figure 5:**
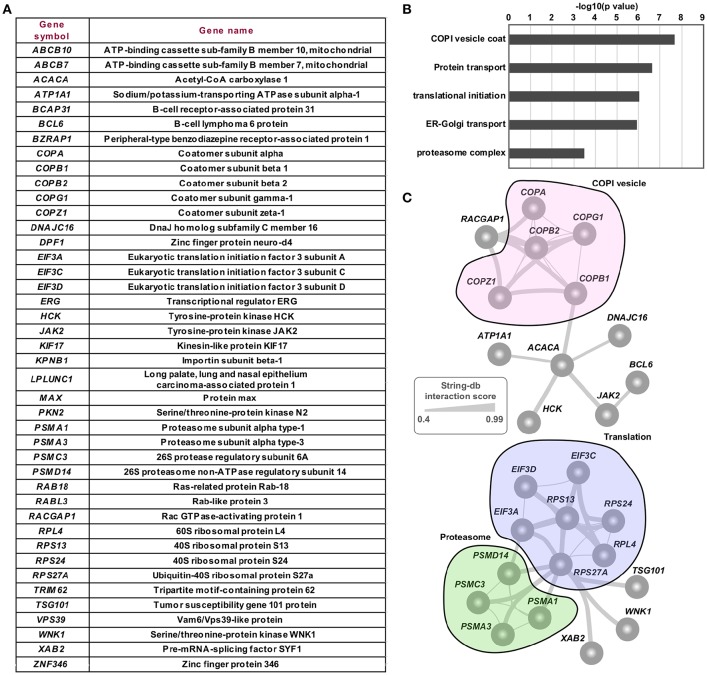
Integration of hvPPI with RNAi screen. **(A)** Top proviral genes from RNAi screens that are also targeted by multiple viral proteins. **(B)** Barplot showing the significantly enriched cellular processes involving the top targeted and proviral host genes. **(C)** Network view of top targets and their functional relevance.

RNAi screens have been a powerful high-throughput method to identify various cellular functions, including for identification of host restriction factors of viruses (69). In order to explore the functional relevance of the host targets in the hvPPI, we integrated it with five published RNAi screens that performed genome–wide or druggable-genome–wide RNAi screens for identifying host factors of HCV (41), HPV18 (43), HPV16 (44), SV40 (46), and VACV (45). We found that host targets from the hvPPI were spread across the spectrum of genes with proviral as well as antiviral phenotype (Figure S5), thus showing that targeting of the host protein by the virus could lead into any direction that favors the virus. We then investigated the top 50 proviral genes that are also targeted by the viral proteins as seen in the hvPPI. We identified 42 host proteins (Figure 5A) that were significantly enriched for coatomer protein complex 1 (COPI), protein translation/transport and proteasome (Figure 5B). This further substantiates the findings from the earlier section on the core cellular processes targeted by the viruses. Network analysis of these top hits showed high level on connectivity and crosstalk—for example between the translation and proteasome machinery (Figure 5C). Vesicle carriers are involved in the transport of membranes and proteins. COPI system is one of the three vesicular carrier systems that is involved in the early secretory pathway (70–72). Moreover, it has been pointed out that there is a strong similarity between vesicular transport and viral transport [viral entry to budding process, (73)]; thus making COPI system important for the viral life cycle. In addition to the findings of the present study, siRNA-based silencing of COPI lead to a decrease in entry and subsequent gene expression of IAV, VSV, LCMV, and HPIV3 and disruption of the COPI complex inhibited the production of infectious progeny virus (74, 75). COPI coatomer inactivation results in a direct decrease of VSV attachment and uptake, but not for membrane fusion or RNP release; however the direct mechanism remains unclear (76). Altogether, these top hits including the COPI system could serve as targets for developing therapeutic antiviral intervention strategies for a broad group of viruses.

### 3.6. Combining Host-Virus and Drug-Gene Interactions Reveals Novel Activities of Broad-Spectrum Antiviral Agents Against Hepatitis C Virus and Human Metapneumovirus

Our analyses pointed out that viral evasion mechanism observed in one virus could also be relevant for other viruses. To test this, we obtained known drug-gene interactions from DGIdb (77).

We selected 28 investigational/experimental/approved antivirals compounds (30) which had DGIdb annotated targets that are part of the hvPPI. We added 12 agents as controls (Table S1 and Figure S6). We tested 40 broad-spectrum-antivirals against GFP-expressing human metapneumovirus (HMPV) NL/1/00 strain (78). Seven different concentrations of the compounds were added to HMPV or mock-infected cells. HMPV-induced GFP expression and cell viability was measured and after 48 h to determine compound efficiency and toxicity. After the initial screening, we identified five compounds, which lowered GFP-expression without detectable cytotoxicity (with SI > 3). We repeated the experiment with these compounds. The experiments revealed novel activity of azacytidine, lopinavir, nitazoxanide, itraconazole, and oritavancin against HMPV (Figure S7A and Table 1, Table S2). Similarly, we examined toxicity and antiviral activity of broad-spectrum-antivirals against GFP-expressing HCV in Huh-7.5 cells using previously described procedures (49). Our test identified azithromycin, cidofovir, oritavancin, dibucaine, gefitinib, minocycline, and pirlindole mesylate as novel anti-HCV agents with SI > 3 (Figure S7B and Table 1, Table S2). In summary, our meta-analysis approach of the hvPPI could provide novel and faster approaches for the re-purposing of existing drugs as antiviral agents.

**Table 1:** Characteristics, half-maximal cytotoxic concentration (*CC*_50_), the half-maximal effective concentration (*EC*_50_), and minimal selectivity indexes ($SI=\frac{CC_{50}}{EC_{50}}$) for selected broad-spectrum antivirals.

**Compound**	**ChEMBL ID**	**Approved use**	**Virus**	**Cell line**	**CC_50_ [μ M]**	**EC_50_ [μ M]**	**SI**
Azithromycin	529	Antibiotic	HCV	Huh-7.5	<30	>10	>3
Cidofovir	152	Anti-CMV	HCV	Huh-7.5	>30	<1	>30
Dibucaine	1086	Local anesthetic	HCV	Huh-7.5	11.6	1.4	8.4
Gefitinib	939	Anticancer	HCV	Huh-7.5	11.6	1.1	10.8
Minocycline	1434	Antibiotic	HCV	Huh-7.5	11.6	5.2	>5.7
Oritavancin	1688530	Antibiotic	HCV	Huh-7.5	>30	<3	>10
Pirlindole mesylate	32350	Antidepressant	HCV	Huh-7.5	>30	<10	>3
Azacitidine	1489	Anticancer	HPMV	RPE	>50	1.2	41.7
Itraconazole	22587	Antifungal	HPMV	RPE	28.2	5	5.6
Lopinavir	729	Antiretroviral	HPMV	RPE	29.7	3.6	8.3
Nitazoxanide	1401	Antiparasitic	HPMV	RPE	>50	2.6	>11.5
Oritavancin	1688530	Antibiotic	HMPV	RPE	>50	2.6	>11.5

*The measurements were repeated three times (p < 0.05)*.

## 4. Discussion

Using integrative analysis of orthogonal datasets our study provides a comprehensive view of viral evasion mechanisms.

In particular, our analysis of the hvPPI network revealed that all the viruses have evolved to target proteins that are central and have strong control over the human interactome. Host proteins targeted by viruses contain a high proportion of intrinsically disordered regions. We identified the core cellular processes and associated proteins that are targeted by all viruses. Detailed comparative analysis of the subcellular localization of the host proteins showed commonality and specificity both between viral proteins from different strains of the same virus; and between viruses. Integrating hvPPI with functional RNAi screens showed that 28% of the hvPPI are host factors of one or more virus. hvPPI data-based drug re-purposing screen identified novel activities for various broad-spectrum antivirals against HMPV and HCV.

This unique dataset can be used for further detailed interrogation of the mechanisms behind viral evasion. This could serve as a starting point for identifying novel host targets and generating hypothesis in the context of viral evasion and development of pan-viral therapeutic intervention strategies. The methods described here also provide unique ways of dissecting the orthogonal datasets. Various analyses from this study have highlighted the existence on pan-viral evasion points that could be utilized for the development of host-directed antiviral therapies. It is also intriguing to see that there is commonality and specificity at the level of sub cellular localization of the viral targets. Our analyses have underlined some salient features in the context of IAV, HPV, DENV, and HCV. Further detailed analysis in this context along with protein sequence features, such as Short Linear Motifs [SLiMs; (79)] would provide novel insights as well as deeper understanding of how small sequence features are involved in the hijacking of the host machinery. Integration of such data with known drug-gene interactions provides a clear estimate of the druggable proportion in the hvPPI. Our meta-analysis approach of the hvPPI could provide novel avenues of re-purposing existing drugs for antiviral targeting strategies.

Our meta-analysis approach of the hvPPI could provide novel avenues of re-purposing existing drugs for antiviral targeting strategies. To prove the concept, we tested 40 BSAs against HMPV, HCV, Sindbis virus (SINV), cytomegalovirus (CMV), and hepatitis B virus (HBV). Importantly, 28 BSAs have DGIdb annotated targets that are part of the hvPPI, whereas 12 were used as controls. These safe-in-man drugs have already been used as investigational agents or experimental drugs in different virus infections (Table S2). We demonstrated novel antiviral effects of azacytidine, itraconazole, lopinavir, nitazoxanide, and oritavancin against HMPV, as well as cidofovir, dibucaine, azithromycin, gefitinib, minocycline, oritavancin, and pirlindole against HCV.

Azithromycin, is an FDA-approved antibiotic of the macrolide family. It is also an investigational agent against RSV and experimental agent against EBOV, HRV-A, ZIKV, and RSV. Cidofovir is an FDA-approved anti-CMV drug. It is also investigational agent against AdV, BKV, HPV, HSV-1, HSV-2, and experimental drug against B19V. Dibucaine is an FDA-approved amide local anesthetic. In addition, it is experimental anti-HEV-A, HEV-B, HEV-D, and EBOV agent. Gefitinib is an FDA approved anticancer drug. It has also antiviral activity against BKV, CMV, and VACV. Minocycline is a broad-spectrum antibiotic and experimental anti-DENV, HIV-1, and WNV agent. Oritavancin is a semisynthetic glycopeptide antibiotic used for the treatment of Gram-positive bacterial skin infections. It also inhibits EBOV, MERS-CoV, and SARS-CoV infections. Pirlindole is an antidepressant, which is also experimental anti-HEV-A, HEV-B, and HEV-D agent. Azacitidine is a chemical analog of cytidine, which is used in the treatment of myelodysplastic syndrome. It is also an experimental anti-AdV, FLUAV, RVFV, HIV-1, and HIV-2 agent. Itraconazole is an antifungal medication. It is also used as experimental anti-HEV-B, HRV-B, HRV-A, Par-A3, and SAFV agent. Nitazoxanide is a broad-spectrum antiparasitic drug, which is also investigational agent against FLUAV and HCV and experimental anti-CHIKV, RSV, HBV, HIV-1, VACV, RV, JEV, MERS-CoV, NoV, RuV, and ZIKV agent. Lopinavir is an FDA-approved antiretroviral of the protease inhibitor class. It is also investigational anti-MERS-CoV and experimental anti-ZIKV agent (Table S2). In addition to inhibition of viral proteases (Table S2), Lopinavir was reported to induce host RNaseL production in infected and non-infected cells (80). RNaseL is endoribonuclease that is a part of interferon (IFN) antiviral response, which is the most critical node of virus-host interactions. Although, the antiviral mechanisms of action of other compounds are still unknown, these agents could inhibit steps of viral infections, which precede reporter protein expression from viral RNA.

In summary, our results indicate that existing BSAs could be re-purposed to other viral infections. To further expand a spectrum of their activities, these BSAs could be tested against other viruses. Re-purposing these and other safe-in-man antiviral therapeutics could save resources and time needed for development of novel drugs to quickly address unmet medical needs, because safety profiles of these agents in humans are available. Effective treatment with broad-spectrum-antivirals may shortly become available, pending the results of further pre-clinical studies and clinical trials. This, in turn, means that some broad-spectrum-antivirals could be used for rapid management of new or emerging drug-resistant strains, as well as for first-line treatment or for prophylaxis of acute virus infections or for viral co-infections. The most effective and tolerable compounds could expand the available therapeutics for the treatment of viral diseases, improving preparedness and the protection of the general population from viral epidemics and pandemics.

## Data Availability

All datasets used for this study are accessible as stated in the Materials and Methods section 2.1.

## Author Contributions

RK and KB performed all the bioinformatics and network analysis. AI, TTT, PA, SK, MT, EZ, UD, AV, RC, HK-K, AB, TT, AM, VO, MB, MA, DS, MK, OV, MW, and DK contributed to the drug re-purposing screen. DK supervised the drug re-purposing screen. GS-F and BS provided data. RK conceived and supervised the study. RK, DK, and KB wrote the manuscript. All authors contributed, read, and approved the manuscript.

### Conflict of Interest Statement

The authors declare that the research was conducted in the absence of any commercial or financial relationships that could be construed as a potential conflict of interest.
