# Supplementary material for: Common Nodes of Virus–Host Interaction Revealed Through an Integrated Network Analysis
Source: Front Immunol. 2019 Oct 4;10:2186. doi: 10.3389/fimmu.2019.02186 (PMC6787150; doi:10.3389/fimmu.2019.02186)
Supplement: Supplementary file 2 [file Data_Sheet_1.PDF]

Supplementary Material

1 SUPPLEMENTARY TABLES AND FIGURES

1.1 Figures

A)

Virus	Genome type	Number of baits	Experiment type	Number of interactions	Source
Adeno-associated virus 5 (AAV5)	dsDNA	7	AP-MS & Y2H	154	Rozenblatt-Rosen et al., 2012
Dengue virus (DENV)	ssRNA+	8	Y2H	135	Khadka et al., 2011
Epstein-Barr virus (EBV)	dsDNA	56	AP-MS & Y2H	1887	Calderwood et al., 2007; Rozenblatt-Rosen et al., 2012
Influenza A virus PR8 (IAV-PR8)	ssRNA-	11	AP-MS & Y2H	209	Shapira et al., 2009; Pichlmair et al., 2012
Influenza virus Udorn (IAV-Udorn)	ssRNA-	8	AP-MS & Y2H	95	Shapira et al., 2009; Pichlmair et al., 2012
Hepatitis C virus (HCV)	ssRNA+	11	Y2H	466	de Chassey et al., 2008
Human immunodeficiency virus 1 (HIV-1)	RT-ssRNA	16	AP-MS	514	Jäger et al., 2011
Human papilloma virus 5 (HPV5)	dsDNA	2	AP-MS & Y2H	62	Rozenblatt-Rosen et al., 2012
Human papilloma virus 6B (HPV6B)	dsDNA	4	AP-MS & Y2H	504	Rozenblatt-Rosen et al., 2012
Human papilloma virus 8 (HPV8)	dsDNA	2	AP-MS & Y2H	158	Rozenblatt-Rosen et al., 2012
Human papilloma virus 11 (HPV11)	dsDNA	4	AP-MS & Y2H	278	Rozenblatt-Rosen et al., 2012
Human papilloma virus 16 (HPV16)	dsDNA	5	AP-MS & Y2H	257	Rozenblatt-Rosen et al., 2012
Human papilloma virus 18 (HPV18)	dsDNA	5	AP-MS & Y2H	368	Rozenblatt-Rosen et al., 2012
Human papilloma virus 33 (HPV33)	dsDNA	4	Y2H	54	Rozenblatt-Rosen et al., 2012
Merkel cell polyomavirus (MCPyV)	dsDNA	4	Y2H	104	Rozenblatt-Rosen et al., 2012
Simian virus 40 (SV40)	dsDNA	2	AP-MS & Y2H	179	Rozenblatt-Rosen et al., 2012
Vaccinia virus (VACV)	dsDNA	36	AP-MS & Y2H	357	Zhang et al., 2009; Pichlmair et al., 2012

B)

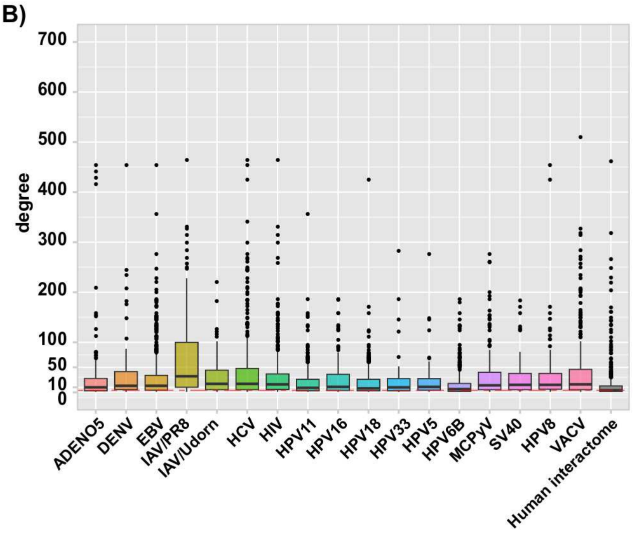

C)

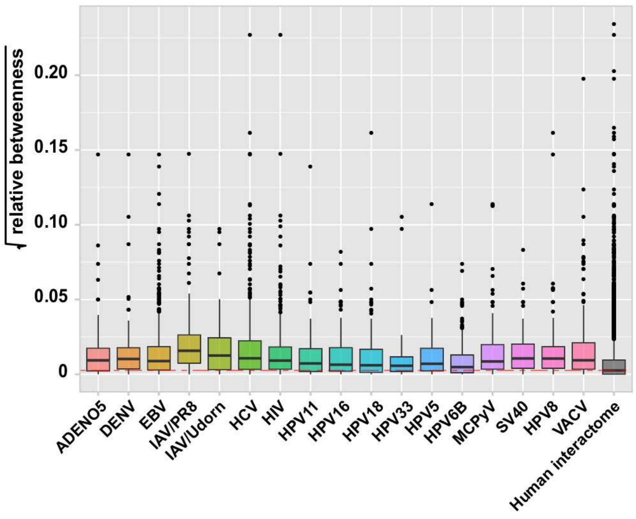

Figure S1. Properties of the hvPPI dataset. **(A)** Table describing the details of the 17 viruses, the genome type, number of baits, experimental type and number of interactions with the host proteins. **(B)** and **(C)** Boxplots showing the distribution of degree and betweenness centrality of targets of each virus as compared to the human proteome.

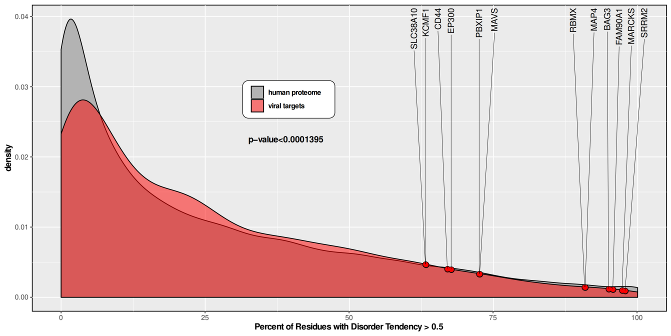

Figure S2. Protein disorder analysis. Density plot of the distribution of host proteins in the hvPPI with percent of residues with disorder tendency greater than 0.5 as predicted by IUPred2A software as compared to the human proteome.

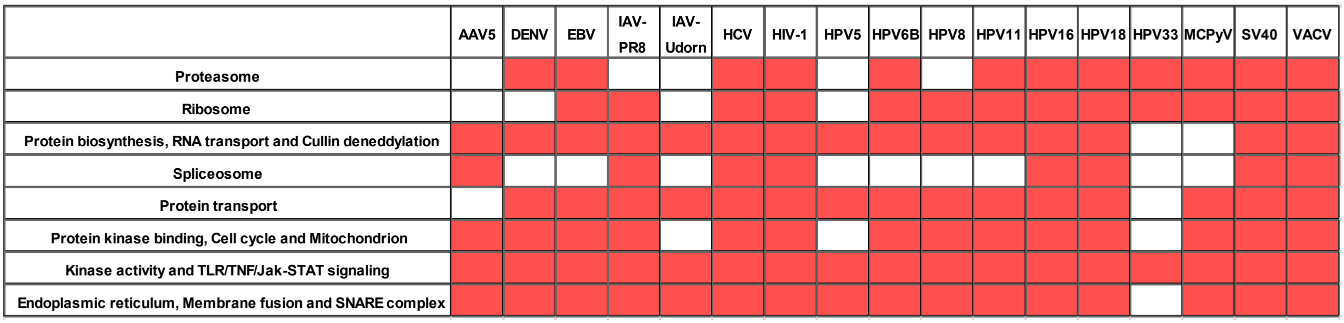

Figure S3. Clusters of hvPPI involved in core cellular processes. Statistically significant cellular processes associated with the host proteins targeted by each virus. Red color shows statistically significant enrichment (p value < 0.01) for that particular virus.

1.2 Tables

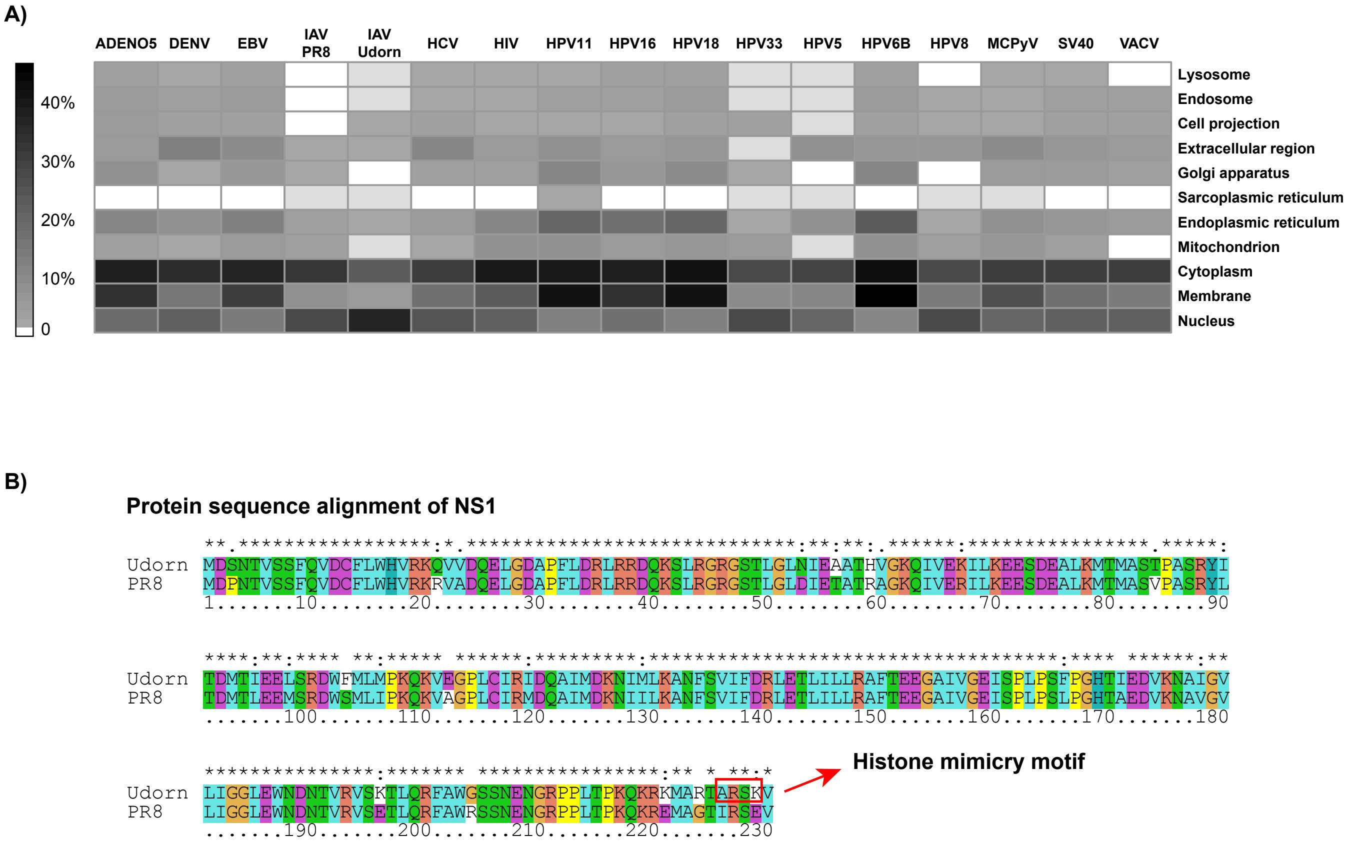

Figure S4. Sub-cellular localisation of the host proteins. **(A)** Heatmap showing the sub-cellular location of the host proteins for the 17 viruses. **(B)** Protein sequence alignment of NS1 proteins from IAV strains PR8 and Udorn.

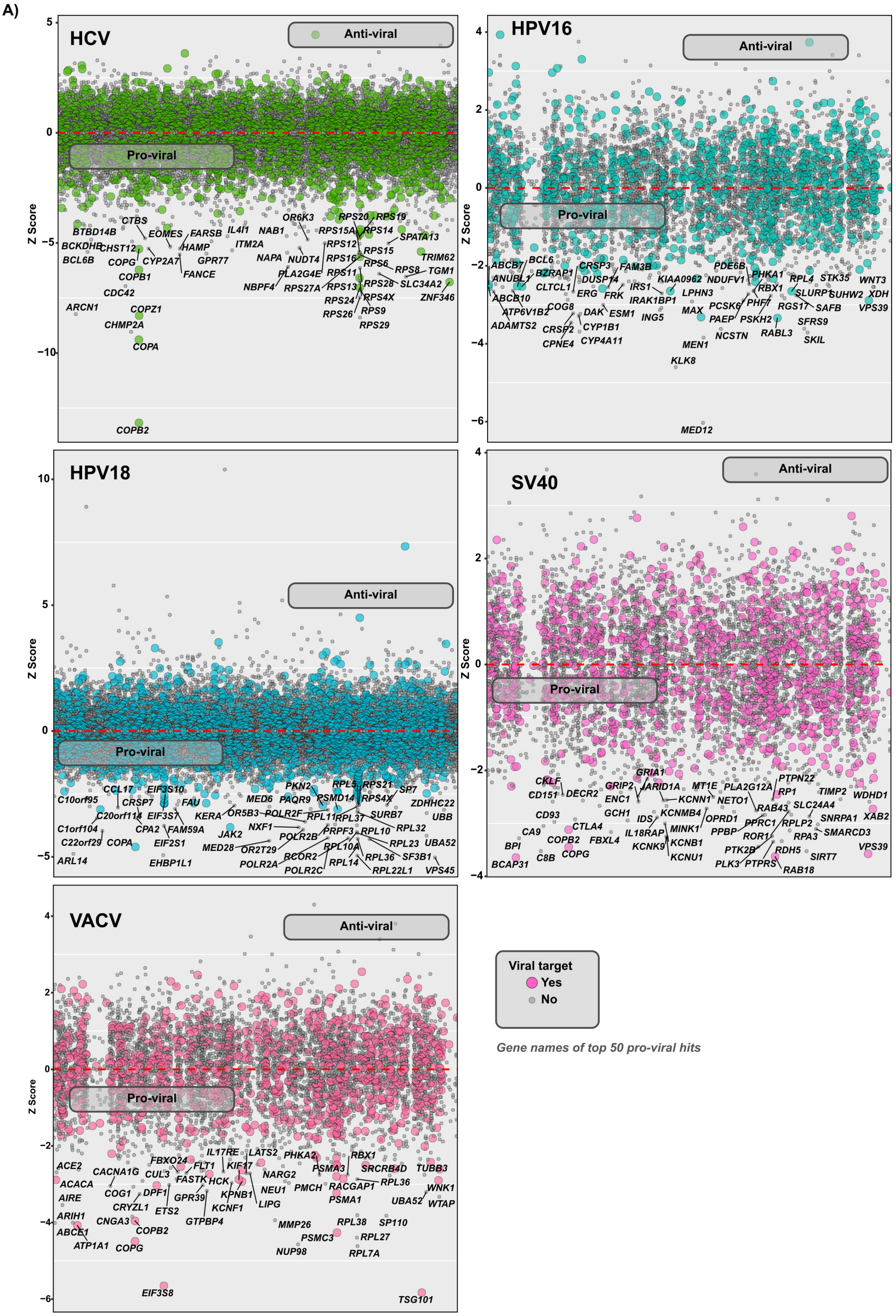

Figure S5. Integration of hvPPI with RNAi screen. Scatter plot showing the viral replication phenotype for 5 different viruses. Gene names for the top 50 proviral genes are highlighted. Genes/Proteins which are part of the hvPPI are shown in bigger dots with colors.

Table S1. Drug re-purposing screen for novel broad-spectrum antivirals. Table showing the tested broad-spectrum antivirals and their representation in the hvPPI network.

Broad–spectrum antiviral drug	# of targets in the host–virus network	# of host-virus interactions
ACYCLOVIR	1	1
ASPIRIN	6	20
AZACYTIDINE	2	2
AZITHROMYCIN	1	7
BORTEZOMIB	47	219
CAFFEINE	2	2
CYCLOSPORINE	15	42
DASATINIB	14	37
DIBUCAINE	1	1
ERLOTINIB	4	16
GEFITINIB	3	11
GEMCITABINE	8	15
HYDROXYCHLOROQUINE	2	9
IMATINIB	7	26
INDOMETHACIN	3	4
IVERMECTIN	1	1
LAMIVUDINE	2	9
LOVASTATIN	5	21
METFORMIN	2	5
MYCOPHENOLICACID	3	28
PENTOSANPOLYSULFATESODIUM	1	1
RIBAVIRIN	1	7
RITONAVIR	1	7
SIMVASTATIN	1	1
SIROLIMUS	14	40
TOPOTECAN	4	16
TRIFLURIDINE	1	5
VERAPAMIL	3	12
ITRACONAZOLE	0	0
ORITAVANCIN	0	0
LOPINAVIR	0	0
NITAZOXANIDE	0	0
CIDOFOVIR	0	0
MINOCYCLINE	0	0
PIRLINDOLEMESYLATE	0	0

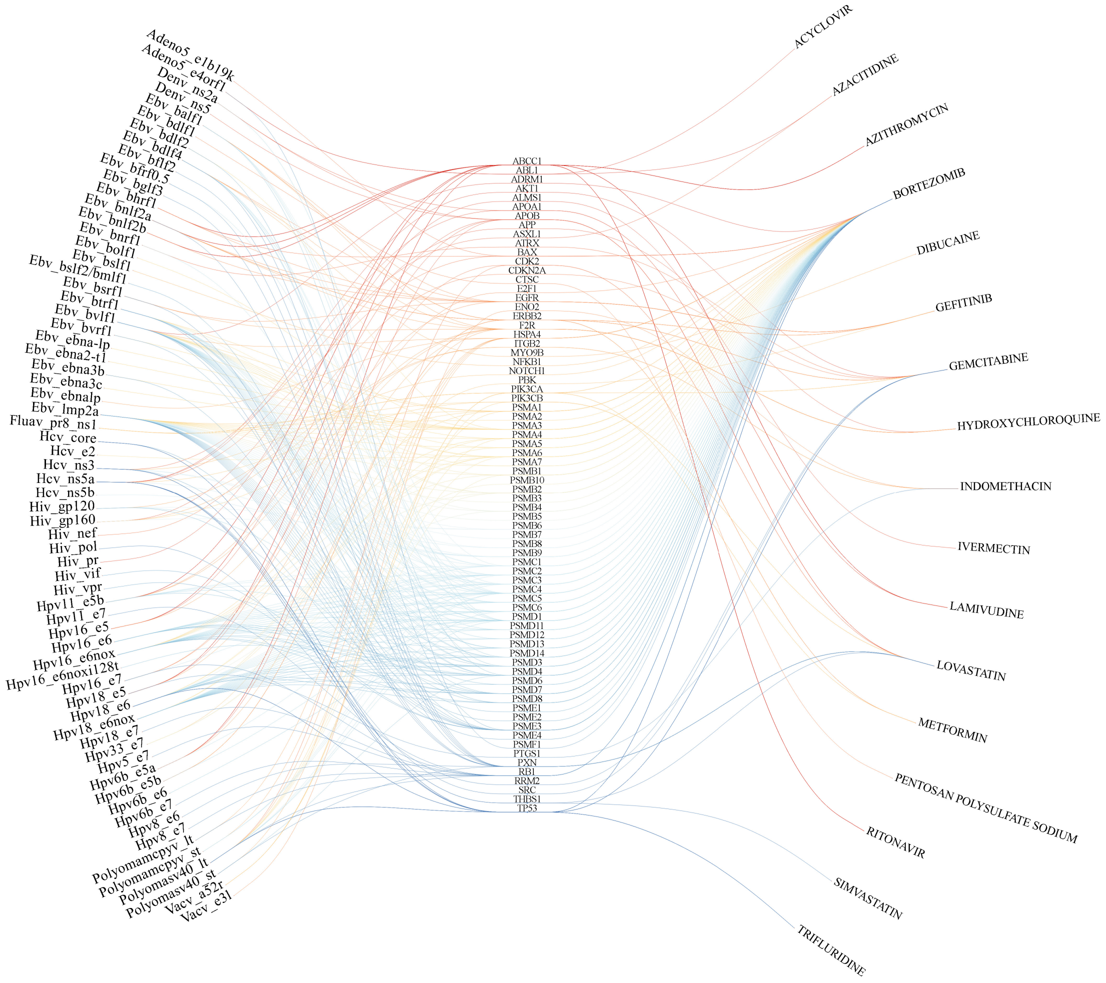

Figure S6. Drug re-purposing screen for novel broad-spectrum antivirals. Interactions between viral and human proteins and known interactions of human proteins with drugs used in this study.

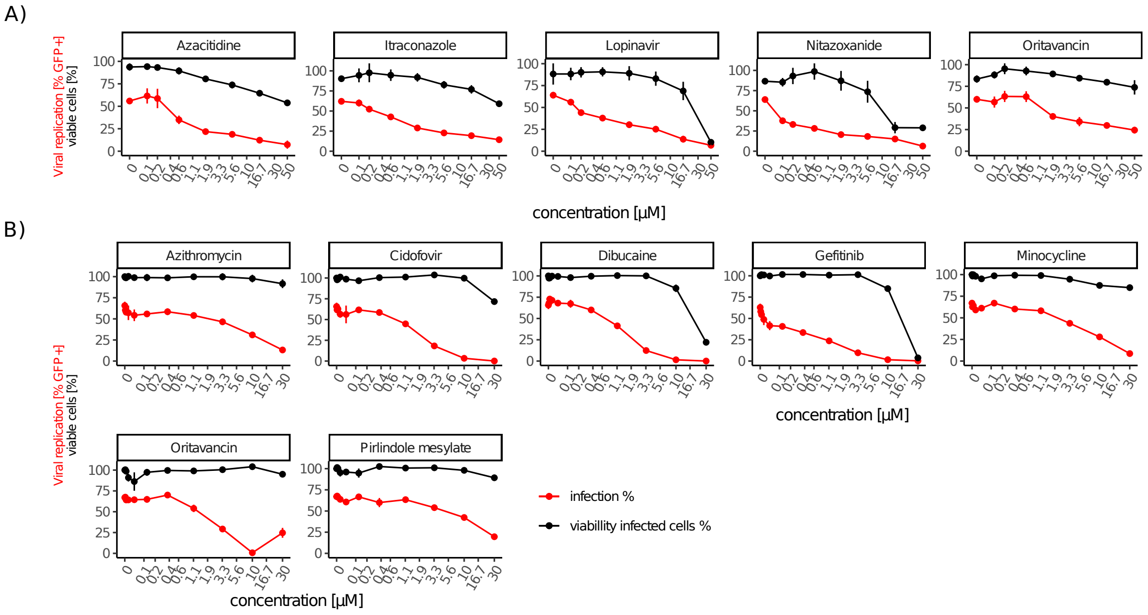

Figure S7. Drug re-purposing screen for novel broad-spectrum antivirals **(A)** At non-cytotoxic concentrations selected broad-spectrum antiviral agents inhibit HMPV-mediated GFP expression in RPE cells. Efficacy of antiviral agents as shown by the percent of GFP-expressing cells decreasing in response to increasing concentration of antiviral agents (Mean ±SD, n=3). Cytotoxicity of antiviral agents as shown by the percent of live cells (Mean ±SD, n=3). **(B)** At non-cytotoxic concentrations selected broad-spectrum antiviral agents inhibit HCV-mediated GFP expression in Huh7.5 cells (Mean ±SD, n=3).
